# Exploring Deep Cervical Compartments in Head and Neck Surgical Oncology through Augmented Reality Vision: A Proof of Concept

**DOI:** 10.3390/jcm12206650

**Published:** 2023-10-20

**Authors:** Alessandro Tel, Marco Zeppieri, Massimo Robiony, Salvatore Sembronio, Shankeeth Vinayahalingam, Antonio Pontoriero, Stefano Pergolizzi, Filippo Flavio Angileri, Leopoldo Spadea, Tamara Ius

**Affiliations:** 1 Clinic of Maxillofacial Surgery, Head-Neck and NeuroScience Department, University Hospital of Udine, Piazzale S. Maria della Misericordia 15, 33100 Udine, Italy; alessandro.tel@icloud.com (A.T.);; 2 Department of Ophthalmology, University Hospital of Udine, Piazzale S. Maria della Misericordia 15, 33100 Udine, Italy; 3 Department of Maxillofacial Surgery, Radboud Medical University, Geert Grooteplein Zuid 10, 6525 GA Nijmegen, The Netherlands; 4 Radiation Oncology Unit, Department of Biomedical, Dental Science and Morphological and Functional Images, University of Messina, 98125 Messina, Italy; 5 Neurosurgery Unit, Department of Biomedical, Dental Science and Morphological and Functional Images, 98125 Messina, Italy; 6 Eye Clinic, Policlinico Umberto I, “Sapienza” University of Rome, 00142 Rome, Italy; 7 Neurosurgery Unit, Head-Neck and NeuroScience Department, University Hospital of Udine, Piazzale S. Maria della Misericordia 15, 33100 Udine, Italy

**Keywords:** head and neck cancer, virtual planning, augmented reality, neck disease, image-guided surgery

## Abstract

Background: Virtual surgical planning allows surgeons to meticulously define surgical procedures by creating a digital replica of patients’ anatomy. This enables precise preoperative assessment, facilitating the selection of optimal surgical approaches and the customization of treatment plans. In neck surgery, virtual planning has been significantly underreported compared to craniofacial surgery, due to a multitude of factors, including the predominance of soft tissues, the unavailability of intraoperative navigation and the complexity of segmenting such areas. Augmented reality represents the most innovative approach to translate virtual planning for real patients, as it merges the digital world with the surgical field in real time. Surgeons can access patient-specific data directly within their field of view, through dedicated visors. In head and neck surgical oncology, augmented reality systems overlay critical anatomical information onto the surgeon’s visual field. This aids in locating and preserving vital structures, such as nerves and blood vessels, during complex procedures. In this paper, the authors examine a series of patients undergoing complex neck surgical oncology procedures with prior virtual surgical planning analysis. For each patient, the surgical plan was imported in Hololens headset to allow for intraoperative augmented reality visualization. The authors discuss the results of this preliminary investigation, tracing the conceptual framework for an increasing AR implementation in complex head and neck surgical oncology procedures.

## 1. Introduction

Surgery in deep neck areas has always represented a challenge, even for the most expert surgeons. The neck is a complex anatomical region, in which multiple vulnerable structures intersect each other, and dissection has to be carefully accomplished to avoid any possible injury. Disease processes occurring in such spaces require careful surgical planning, in which the most direct and harmless form of access needs to be defined [1].

Technology can enhance the study of the case through advanced imaging modalities that can be used to perform virtual surgical planning, in which a digitalized patient anatomy is created to represent a computational replica of the tumor surrounded by critical anatomical regions. The definition of hard and soft tissue anatomy in proximity to the tumor can provide informative clues to define the most suitable surgical approach, while at the same time privileging, when possible, the preservation of noble structures.

Although, nowadays, virtual surgical planning has been extensively described across the literature, its applications in soft tissue reconstruction and especially in the neck region are considerably underreported, owing to the difficulty of performing the segmentation of anatomy in such a complex anatomical region that is conventionally studied using CT scans. Thus, a multi-modality imaging technique is needed to investigate soft tissue compartments and perform an accurate segmentation to yield a reliable representation of anatomy, as described by the same authors [2].

In addition, translating virtual surgical planning in neck surgery has always represented a major problem, with few solutions reported over the years. Navigation has been extensively described in the craniofacial region, due to stable and widespread skeletal landmarks, which lead to a less deformable anatomy, but its use in the neck region raises major issues in terms of navigator calibration, as well as a high deformability of the operative field [3]. Similarly, while surgical guides represent one of the key tools to connect virtual surgical planning to the real patient in bone surgical procedures, they do not find any application in the neck region, where soft tissue is predominant [4]. Moreover, surgical guides fail to provide the surgeon with a reliable system to check the depth of dissection, as they are exclusively conceived to translate the design of a virtual osteotomy in real procedures. On the other hand, navigation is expensive, requires a cumbersome apparatus in the OR and needs the constant feedback of the pointer to check the depth and localize structures.

Augmented reality (AR) is seen as one leading technological solution that will represent a “game-changer” approach in the future for computer-aided vision. The possibility to superimpose a virtual patient replica to the real operative field without being restricted to the acquisition of physical landmarks makes it suitable for anatomical regions that cannot be approached using traditional navigation stations [5]. AR is a dynamic technology and can be efficiently coupled with wearable devices, enabling a decrease in the need for physical space in the OR, as well as decreasing costs and enabling surgeons to constantly check that the virtual plan made corresponds with the real patient.

The purpose of this paper is to introduce a novel protocol for the preoperative digitalization of neck anatomy in patients with neck masses and describe an AR-based workflow to speculate on how AR will impact neck surgical oncology. Despite AR being immature and certainly unreliable at this stage, a practical demonstration of its potential may open new insights and accelerate the development of more accurate solutions in this surgical field.

## 2. Materials and Methods

### 2.1. Patient Selection

This is a retrospective observational study conducted from February 2022 to July 2023. Five patients were enrolled with disease processes located in the deep cervical spaces requiring surgical excision. The application of AR was not used to guide surgical excision, as it is still unreliable; however, the system was tested intraoperatively for calibration, ergonomics and correspondence with the real field. This study was conducted according to STROBE guidelines and the Helsinki declaration, and it was approved with the following number: protocol IRB_45_2020 approved by the Institutional Review Board (IRB) of the University of Udine.

### 2.2. Creation of the Virtual Patient

#### 2.2.1. Imaging Acquisition Protocols

The protocol for digital patient reconstruction depends on strict imaging protocols that need to be acquired based on the anatomical structure that is to be segmented. Bone tissue is appropriately studied using CT scan with ultrathin slices (at least 0.625 mm) and a 512 × 512 px matrix to enhance spatial resolution.

Tumors are generally well studied using gadolinium-enhanced MR in VIBE sequence with volumetric isotropic acquisition (1 mm voxel at least) and a 512 × 512 px matrix. A panoramic, volumetric neuronavigator acquisition protocol (NNV) can account for the segmentation of surrounding muscles and other soft tissue anatomy, as the VIBE sequence is generally restricted to a limited field. Arteries are acquired using a time-of-flight volumetric sequence with 0.3 mm slice thickness in the intracranial space and proximal cervical space. Similarly, 3D MR venography can define venous vessels up to a plane crossing the carotid bifurcation.

#### 2.2.2. Segmentation and Virtual Surgical Planning

Segmentation of medical images is performed using the software Materialise Mimics v. 25 (Materialise, Leuven, Belgium). Bone anatomy is easily detected using threshold-based methods by setting the bottom limit to the bone density Hounfield Unit (HU) range. Vessels are most easily tracked using methods of dynamic region growing, in which contiguous voxels are sampled and defined by spatial proximity criteria. Tumor mass and muscles are reliably defined across their boundaries using manual artificial intelligence (AI)-guided smart brushes that enable the clinician to paint the tumor lesion of the muscle group. Once masks have been accurately created for each object of interest, they are retesselated to be converted into high-detail polymeshes, usable for virtual surgical planning.

**Figure 1:**
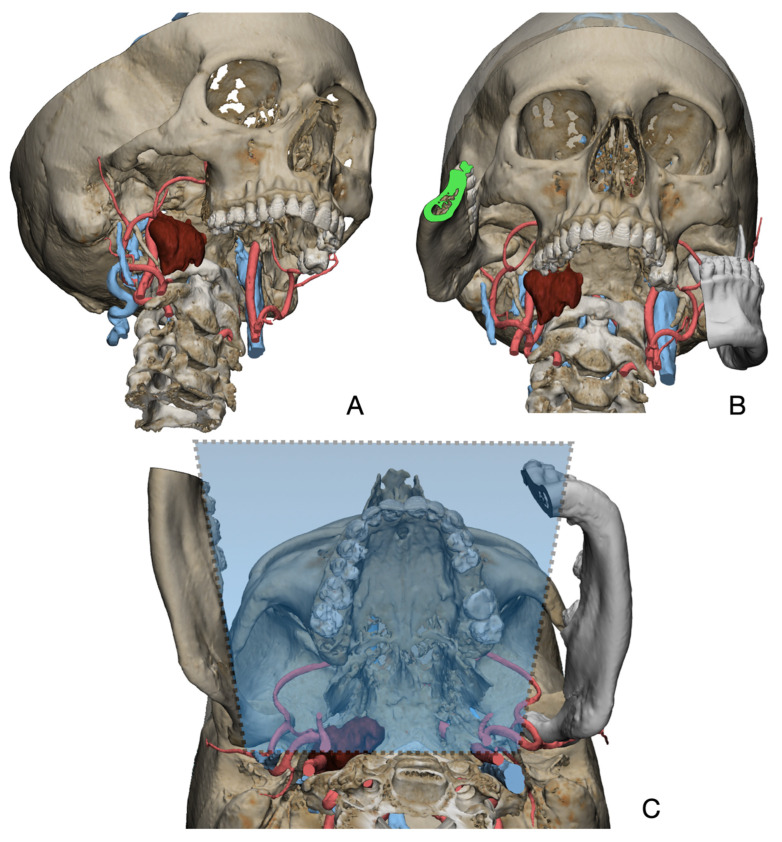
Virtual surgical planning to study the resection of a tumor arising in the upper cervical tract. (**A**) multilayer anatomical segmentation and reconstruction of a patient-specific anatomical atlas; (**B**) simulation of mandibulotomy with a mandibular swing approach; (**C**) definition of the access portal through the mandibular swing approach.

In Materialise 3-matic, a mandibular osteotomy is simulated, taking into account the position of teeth roots located in the most favorable site to allow the mandibular swing approach to optimally expose the site of disease. The full mandibular swing movement is simulated to define the optimal access portal to the cervical region in case of disease located in the upper neck region (Figure 1). For disease located in the lower neck region, the mandibular osteotomy is not necessarily simulated.

### 2.3. Surgery

**Figure 2:**
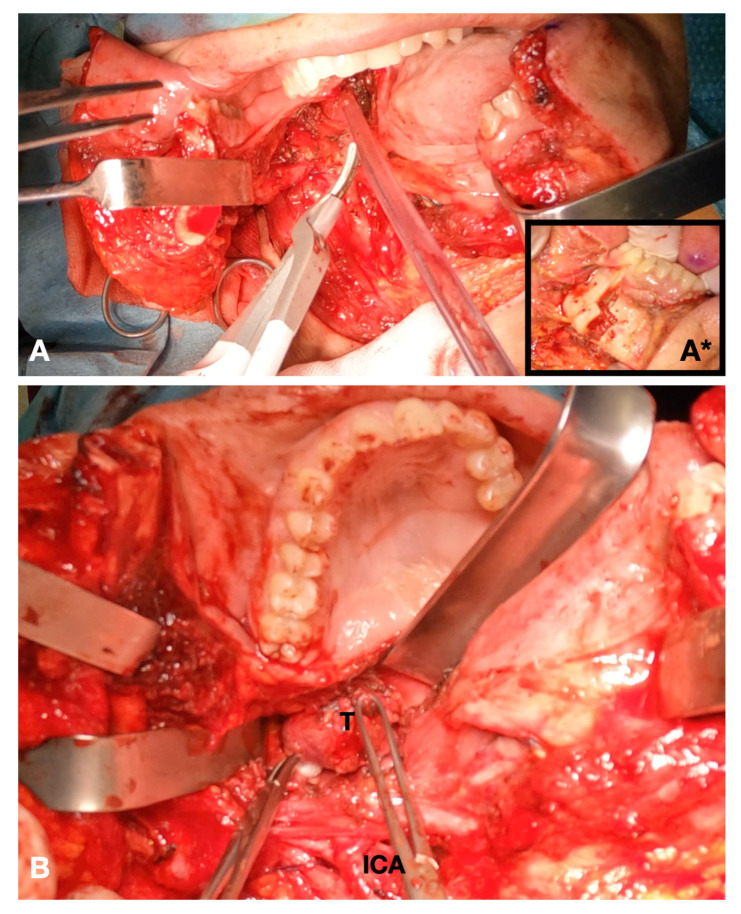
Intraoperative view. (**A**) Definition of the mandibular swing approach; insert (A*) piezosurgical mandibulotomy; (**B**) exposure of the tumor.

A cervicotomy was designed, and a myocutaneous flap was raised. The anterior margin of the sterneocleidomastoid muscle was isolated, and dissection was carried out to identify and protect the spinal nerve. The posterior belly of the digastric muscle was localized to assess the plane of dissection. Depending on the location of the disease, two different approaches were used:

- For disease in the upper cervical tract, the inferior mandibular border was exposed in correspondence with the mandibular symphysis and parasymphyseal region. Incision of the mandible fornix continuing to the lateral mouth floor was performed to enable rotation of the hemimandible on the condylar pivot. During the sectioning of the lateral oral floor, attention was carried out to preserve the lingual nerve. Prebending and predrilling of screw holes for two osteosynthesis titanium plates were performed prior to completing the mandibular splint. A paramedian osteotomy between the roots of the canine and first premolar was accomplished by means of piezosurgical cut. The mandible was extrarotated on the disease side, and the mylohyoid muscle was further sectioned to fully widen the surgical access to the deep cervical neck compartments (Figure 2). After the mass was removed, the mandible was reconstructed using the premodeled titanium plates. Multilayer suture of the neck and intraoral mucosal suture of the mouth floor were completed.
- For disease in the middle or lower cervical tract, the transmandibular approach was not generally deemed necessary. In this case, the surgical approach considered the steps for the traditional neck dissection surgery.

### 2.4. Augmented Reality

**Figure 3:**
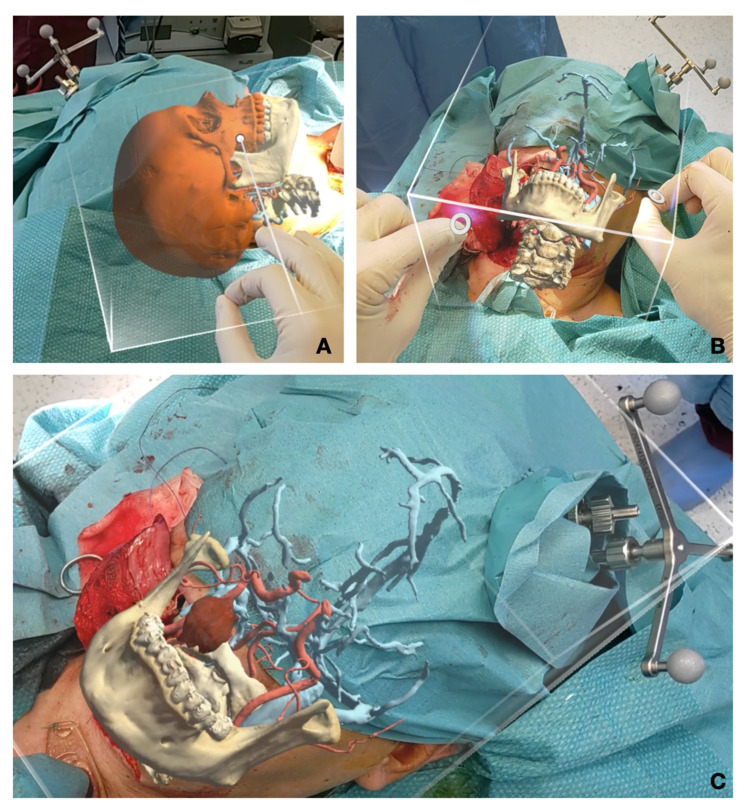
Intraoperative augmented reality captures. (**A**) Calibration and fine tuning of the AR image using object tracking and gestures before incision; (**B**) intraoperative calibration after the transmandibular swing approach; (**C**) correctly repositioned hologram to drive tumor identification during surgery.

**Figure 4:**
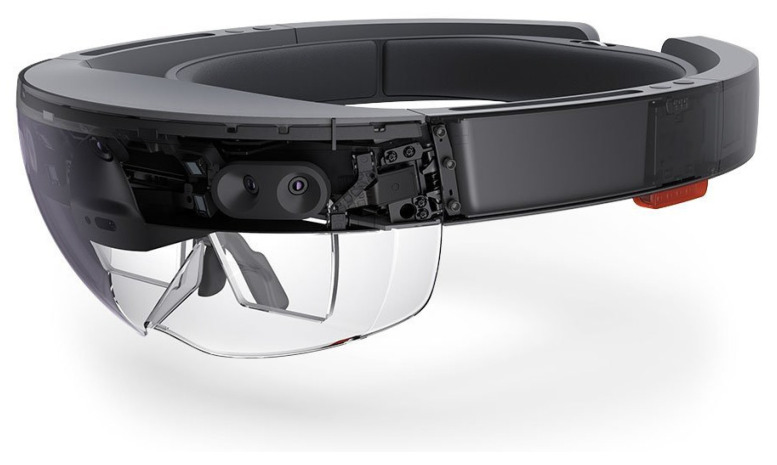
Appearance of Hololens 2 headset and view of the internal sensors for spatial recognition and infrared cameras.

STLs of surgical planning were previously exported and uploaded in Materialise Mimics Viewer, an online application that enables the creation of AR projects for holographic visualization of surgical planning (Figure 3). The same application can be installed in Microsoft Hololens 2.0 (Microsoft, Redmond, WA, USA) operating system (Windows Holographic Operating System), in order to make the project available for the Hololens headset. Hololens includes an efficient technology for hand tracking, allowing the user to physically interact with the AR hologram using gestures. Moreover, infrared stereocameras are installed at both sides of the headset to map the spatial environment, allowing for the stable maintenance of the AR image in the real space (Figure 4). The fluidity of the AR image is improved thanks to eye-tracking technology that stabilizes the object in relation to the user’s eye movements. A preliminary calibration is performed before applying sterile drapes to maximize the visible anatomy. Calibration is further refined intraoperatively to adapt it to corresponding anatomy and head movements required by the surgical procedure.

As AR is still unreliable and not validated for precision surgery yet provides highly improved orientation and understanding capabilities, it was coupled with intraoperative navigation of surgical planning, to enhance lesion targeting and anatomical structure identification.

### 2.5. Literature Review

The authors conducted a narrative literature review to assess the presence of virtual surgical planning and augmented reality in head and neck surgical oncology. For this purpose, a dedicated research query was formulated as follows: ((“augmented reality” OR “AR”) AND (“head and neck” OR ENT OR maxillo*) AND (oncolog*) AND (surgery OR surgical)). The syntax was modified to make it suitable for each of the following databases: Pubmed/Medline, Embase, ScienceDirect, Ovid. Moreover, a broad-spectrum search strategy was implemented in Pubmed/Medline using MeSH terms based on the following string: (“Augmented Reality” [Mesh]) AND “Head and Neck Neoplasms” [Mesh]. Criteria for paper selection included: publication no older than 2018; application in head and neck surgical oncology; clinical application in patients within the OR; English; reported pictures of the AR system; presence of detailed surgical planning. Exclusion criteria were as follows: applications outside head and neck region; application in fields other than surgical oncology; in silico or in vitro studies without the clinical implementation in real surgical scenarios; languages other than English; omission of pictures showing AR applied in the surgical field; no virtual surgical planning presented.

## 3. Results

Five patients were recruited in the present study. The mean age was 69 years old. In two patients, it was not possible to acquire the dedicated MR protocol, and surgical planning was performed using CT angiography, involving inferior tumor detail and the loss of separate arterial and venous vasculature reconstruction. Table 1 reports patient details, including virtual reconstruction, disease location and etiology, imaging protocol and AR support.

For all patients, AR was applied. AR was applied in combination with navigation only in cases of disease localized in the upper cervical tract, due to the possibility of performing neuronavigational calibration. For disease localized exclusively in the neck, navigation was not applicable. All surgeries were successful and uneventful. We report sensitivity loss of the lateral tongue ipsilateral to the mandibular swing side due to lingual nerve sacrifice to widen the surgical corridor through mylohyoid muscle sectioning. There were no major bleeding events.

In all patients, the preoperative virtual definition of an access corridor was related to the improved exposure of the tumor mass during surgery.

The results of our literature review yielded 229 results overall; after applying a threshold for publication year set at no older than 2018, the results were restricted to 164. MeSH tree search yielded 10 results. Combined, the initial search was conducted across 174 papers. A step-by-step selection according to the scheme implemented in PRISMA guidelines, including sequential duplicate removal, title screening, abstract screening and full-text reading, ended up with three studies being selected and carefully analyzed. Two papers deal with oncology-respective procedures, whereas one deals with fibula-free flap harvesting facilitated by AR for reconstructive purposes. The results of paper selection are reported in Table 2.

## 4. Discussion

The use of AR in head and neck surgical oncology has seldom been reported. In the literature, AR has been explored mostly for research purposes, and the authors identified at least three main themes:

- AR explored in terms of computer science and computation: this field mostly includes in silico studies that investigate novel strategies to improve AR usability and efficiency for clinicians [8,9].
- AR compared to other image-guided surgery techniques: such studies compare the advantages and accuracy of novel AR devices and technologies to the current gold standard in head and neck surgery, represented by intraoperative navigation [5,10,11].
- AR used in a real clinical setting: such studies represent the focus of this paper, and very few examples are available to denote the usability of AR for real surgeries [6,12,13].

Head and neck surgical oncology requires a complete preoperative anatomical study and intraoperative guidance, owing to the inherent complexity of this anatomical district and the immediate implications that oncologic resection has in terms of compromising important functions, such as breathing, speaking and swallowing.

In malignant pathology, where surgical resections need to be tailored to individual patients trying to balance surgical radicality and subsequent comorbidities, a careful preoperative study based on virtual surgical planning is mandatory to assess resection margins, noble structure sparing and to configure the most straightforward path to the lesion. The importance of reconstructing a digital anatomical atlas of each patient, including as many segmented structures as possible, enhances the possibility of simulating surgery and foreseeing criticalities. While 3D printing has been proven by a number of studies to facilitate anatomical understanding and replicate surgical maneuvers in advance, it provides only indirect guidance during operative phases [14]. A 3D-printed model can be scoped and manipulated before the surgical act, but it will not allow for direct feedback during surgical motion. Notably, there are highly advanced technologies nowadays, including PolyJet^®^, that enable the simultaneous deposition of different materials within the same print, allowing the manufacture of models with different colors and consistencies to replicate the heterogeneous composition of human tissues [15].

Intraoperative navigation was primarily developed for a neurosurgical setting and relies on a rigid body registration to locate a probe on the patient’s imaging while it is traced by an infrared light of a magnetic field. Subsequent studies, initially led by Schramm and Gellrich, translated this technology into craniofacial scenarios, where it was used to navigate deep spaces of the splanchnocranium, including the skull base, the orbital region, pterygopalatine and infratemporal fossa [16,17,18]. This technology is severely limited when mandibular resections are involved, as it requires a double-tracking motion device to simultaneously register the head and the mandible, which is movable in relation to the skull. However, the main limitation arises in neck compartments, where image guidance might be helpful, especially in locating deep disease processes, as well as pathological lymph nodes and vital structures. Currently, there is no validated methodology to enable image guidance in neck spaces, as navigation is unable to register any imaging sequence below the mandible area. Most importantly, the neck is a highly mobile region, where bending degrees may vary significantly compared to the position in which imaging was originally acquired, thus making the alignment of the preoperative virtual model with the real patient more complex. Therefore, great effort will be required from companies, universities and computer scientists to enable an image-guided system that will bridge a preoperative simulation with the real necks of patient candidates in surgical oncology.

In addition, the field of virtual reality and surgical planning applied in the neck region is considerably underreported, as the segmentation of neck soft structures is more demanding in terms of skills, software equipment and time compared to traditional bone segmentation of the craniofacial region [19]. Among the very few reports, Ignat et al. described a case of parathyroid gland virtual reconstruction, including neck vessels, and laryngeal and thyroid segmentation for virtualization purposes [20], while D’Agostino et al. translated a similar virtual image into a preliminary augmented reality setup but without defining its technical background [21]. Another interesting case was reported by Scherl et al. in a pilot study to assess the use of AR in parotid gland surgery, where virtual models of the parotid and the mandible were overlaid with the real operative field. What this study emphasizes is that the average discrepancy corresponds to 1.3 cm, making this technology still inapplicable to surgery by itself [7]. Such limitations emphasize that AR registration methods are currently too immature to undergo a path of certification and be implemented within routine clinical practice. However, it is important to define their application possibilities as the technology progresses; therefore, the majority of studies are pilot studies or studies defining the boundaries of uncertainty for reliable registration.

In this paper, the authors tried to apply currently available technologies in real cases of complex tumor neck disease to identify the advantages that this technology may involve. Surgical planning described in the Materials and Methods section was exported into an STL file and imported into Materialise Mimics Viewer, where a project suitable for the Microsoft Hololens 2 headset was generated. The headset is not heavy and can be well tolerated by the surgeon during the procedure; however, it is used exclusively in phases that require AR, such as the preliminary surgical access, to enhance incision positioning, and during dissection, to facilitate intraoperative orientation and mass localization, as well as the avoidance of crucial structures, including great vessels. Current systems that lack depth accommodation tend to provoke eye fatigue, an issue that is well known in AR optics, and might be overcome by innovative lenses with multiple focal planes. This system allowed for good ergonomics during surgery, as Hololens-embedded cameras were able to capture the gesture motion of the operator wearing the headset, enabling the fine manipulation of the AR object that could be interactively repositioned, scaled and rotated according to the different spatial perception in every phase. While AR is not a mature technology and, thus, it cannot be granted any certification for medical use, such preliminary investigations are important to facilitate surgeons’ understanding of the potential benefits of this technology applied to neck surgical oncology, including the ability to locate the optimal site to perform an incision into the skin and scoping vulnerable structures before they are dissected and exposed. Conspicuous research is currently being conducted in this field to decrease the mismatch between the virtual hologram and the physical patient, including the implementation of eye-tracking technology and multiple focal planes, which is supposed to add depth to the virtual image, so that its three-dimensional superimposition is balanced with the real focal distance of the eye’s operator, reducing eye fatigue as well [22].

Moreover, AR enables the temporary detachment of the virtual hologram from the registration with the patient and allows us to move it around the physical space to scope the virtual reconstruction separately, from multiple perspectives, enabling surgeons to dive within the patient’s anatomy as if they were manipulating a 3D-printed model.

Another interesting field of research is the use of markerless registration. According to this approach, the AR software installed in the hardware device is able to track the shape of the real object and make it coincide with the virtual 3D model of the surgical planning. Recognition is still impaired by a variety of factors occurring during surgery, such as draping with coverage of broad surfaces that the algorithm might track, as well as light interference, and paucity of stable landmarks, including prominences and depressions. Battaglia et al. described an interesting tracking system based on markerless registration using an AR scene developed within Unity 3D (Unity Technologies, San Francisco, CA, USA) and empowered by Vuforia SDK (software development kit) [5]. Gsaxner et al. tested the application of an instant calibration markerless approach for head and neck surgery that fastens the registration of the virtual planning with the real patient and does not rely on physical fiducials [23]. This technology is based on face detection features and surface reconstruction capabilities that facilitate the coupling of virtually reconstructed surfaces with their real counterparts. Similarly, Tel et al. [13] used the Vuforia engine to enable object tracking in a 3D-printed orbital phantom used for AR-based simulations.

For such a complex and movable structure as the neck, it will become essential to define an “intelligent” system, able to tailor the modifications of head position and cervical spine flexion and extension to corresponding actions translated on the virtual model. For this purpose, mesh-tracking technologies will enable the modification of imported STL, considered as rigid bodies, adapting them to the variability of the surgical scenario. At the same time, such a system will necessarily be based on artificial intelligence (AI) and deep learning (DL), requiring substantial computational power. Dynamic recalibration will become essential to adapt the intraoperative modification of the surgical fields with adaptable surgical planning, also including kinematic sequences, to simulate each procedure and phase as the surgery progresses. The advent of quantum computing will enable the enhancement of existing hardware to an unprecedented extent, enabling AI algorithms to modify the calibration second by second based on the variation in the operative field. AI will substantially improve object recognition capabilities, enabling the capture of a smaller amount of detail to perform the calibration between the real patient and the hologram, which may contribute to ensuring the correct registration when the patient is covered in sterile drapes.

## 5. Conclusions

Despite the underreported use of AR in complex head and neck surgical oncology, this preliminary report shows that this technology has great potential to guide surgeons in the most complex scenarios. The currently available systems enable appropriate ergonomics during surgery but fail to accurately register the real patient with their holographic representation. The use of AR in the operating room is completely different from other applications due to a number of influencing factors, including light interference, patient draping and logistics. We foresee that the advent of AI and self-learning algorithms, together with the advancement of the computational power of new chips, will lead AR to the appropriate accuracy for the clinician and will radically modify intraoperative image guidance.

## Figures and Tables

**Table 1:** Overview of patients recruited for this study, including virtual surgical planning, disease location, imaging protocol and AR device.

Age and Sex	Virtual Surgical Planning	Disease and Location	Imaging Protocol	AR Device
63, F	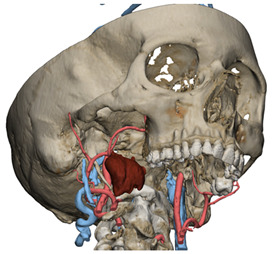	Persistent Schwannoma surrounding ICA	CT MR: VIBE TOF VEN3D NNV	Microsoft Hololens 2
72, M	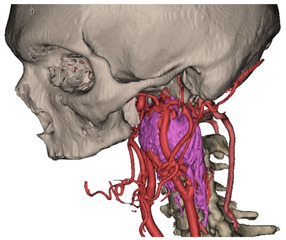	Paraganglioma located at ICA and ECA bifurcation	CT (basal) Contrast-enhanced CT	iPad
59, M	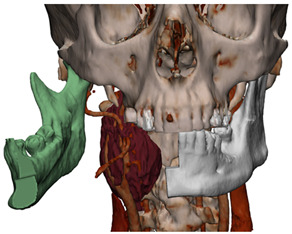	Paraganglioma located at ICA and ECA bifurcation	CT (basal) Contrast-enhanced CT	iPad
76, F	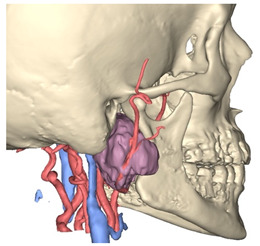	Persistance of deep parotid lobe pleomorphic adenoma	CT MR: VIBE TOF VEN3D NNV	Microsoft Hololens 2
78, M	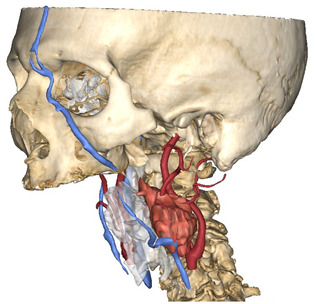	Single cervical metastasis from squamocellular carcinoma of the oral oral cavity	CT MR: VIBE TOF VEN3D NNV	Microsoft Hololens 2

**Table 2:** Papers selected fulfilling all review criteria.

Author and Year	Anatomical Region	AR Hardware	AR Software	Clinical Application	Purpose
[5]	Leg (for reconstructive purposes)	Tablet/Smartphone	Unity 3D	AR assisted fibula flap harvest	Validate a markerless recognition
[6]	Maxillary	Hololens	Unity 3D + Vuforia Engine	Maxillary tumor resection	Validate AR osteotomies in surgical HN oncology
[7]	Parotid	Hololens	Unity 3D; Visual Studio	Intraoperative registration and structure recognition	Pilot study

## Data Availability

The data presented in this study are available on request from the corresponding author.
